# Randomized-controlled phase II trial of salvage chemotherapy after immunization with a *TP53*-transfected dendritic cell-based vaccine (Ad.p53-DC) in patients with recurrent small cell lung cancer

**DOI:** 10.1007/s00262-018-2287-9

**Published:** 2018-12-27

**Authors:** Alberto A. Chiappori, Charles C. Williams, Jhanelle E. Gray, Tawee Tanvetyanon, Eric B. Haura, Ben C. Creelan, Ram Thapa, Dung-Tsa Chen, George R. Simon, Gerold Bepler, Dmitry I. Gabrilovich, Scott J. Antonia

**Affiliations:** 1 Department of Thoracic Oncology, H. Lee Moffitt Cancer Center and Research Institute, 12902, Magnolia Drive, FOB1, Tampa, FL 33612 USA; 2 Department of Biostatistics and Bioinformatics, H. Lee Moffitt Cancer Center and Research Institute, Tampa, FL 33612 USA; 3 MD Anderson Cancer Center, Houston, TX 77030 USA; 4 Karmanos Cancer Institute, Detroit, MI 48201 USA; 5 Wistar Institute, Philadelphia, PA 19104 USA

**Keywords:** Dendritic cell vaccine, Immunotherapy, Small cell lung cancer, *TP53*/p53, Tumor antigens

## Abstract

Small cell lung cancer *TP53* mutations lead to expression of tumor antigens that elicits specific cytotoxic T-cell immune responses. In this phase II study, dendritic cells transfected with wild-type *TP53* (vaccine) were administered to patients with extensive-stage small cell lung cancer after chemotherapy. Patients were randomized 1:1:1 to arm A (observation), arm B (vaccine alone), or arm C (vaccine plus all-trans-retinoic acid). Vaccine was administered every 2 weeks (3 times), and all patients were to receive paclitaxel at progression. Our primary endpoint was overall response rate (ORR) to paclitaxel. The study was not designed to detect overall response rate differences between arms. Of 69 patients enrolled (performance status 0/1, median age 62 years), 55 were treated in stage 1 (18 in arm A, 20 in arm B, and 17 in arm C) and 14 in stage 2 (arm C only), per 2-stage Simon Minimax design. The vaccine was safe, with mostly grade 1/2 toxicities, although 1 arm-B patient experienced grade 3 fatigue and 8 arm-C patients experienced grade 3 toxicities. Positive immune responses were obtained in 20% of arm B (95% confidence interval [CI], 5.3–48.6) and 43.3% of arm C (95% CI 23.9–65.1). The ORRs to the second-line chemotherapy (including paclitaxel) were 15.4% (95% CI 2.7–46.3), 16.7% (95% CI 2.9–49.1), and 23.8% (95% CI 9.1–47.5) for arms A, B, and C, with no survival differences between arms. Although our vaccine failed to improve ORRs to the second-line chemotherapy, its safety profile and therapeutic immune potential remain. Combinations with the other immunotherapeutic agents are reasonable options.

**Electronic supplementary material:**

The online version of this article (10.1007/s00262-018-2287-9) contains supplementary material, which is available to authorized users.

## Introduction

Many malignancies are associated with somatic mutations in *TP53* [1–3], including small cell lung cancer (SCLC), which has an extremely poor prognosis despite frequent initial responses to the standard, first-line, platinum-doublet chemotherapy [4–11]. These mutations often result in increased quantities of abnormal p53 protein in tumor cells [12, 13]. About 90% of patients with SCLC have *TP53* mutations; of these, about 90% over-express mutant p53 protein [14–16]. Several studies have shown that cytotoxic T cells that recognize non-mutant epitopes in p53 can selectively kill malignant cells but not normal cells [12, 13]; therefore, non-mutant p53 may be a reasonable shared antigen for tumor-selective immunotherapy.

One immunotherapeutic strategy that has been developed is the use of dendritic cells (DCs); these potent antigen-presenting cells are “loaded” with target tumor antigens through the use of adenoviral vectors that contain the gene coding for the target antigen [17–19]. We have developed a vaccine consisting of DCs transfected with the wild-type *TP53* gene using an adenoviral vector (Ad.p53 DC vaccine), and the results of our phase II clinical trial designed to test the safety and preliminary efficacy of the Ad.p53 DC vaccine in patients with extensive-stage SCLC were published [20, 21]. Several interesting observations were made at the conclusion of the trial, including (1) the vaccine itself is safe; (2) the vaccine itself produced a partial response (PR) in two patients; (3) a larger-than-expected number of patients treated with chemotherapy following vaccination attained objective tumor regression; (4) tumor antigen-specific T-cell responses were induced by the vaccine in almost half of patients; and (5) when present in patients, myeloid-derived suppressor cells (MDSCs) interfered with the immune response to the vaccine.

Many different factors contribute to the immunosuppressive influence of the tumor microenvironment and suppress anti-tumor immune recognition and response/destruction. MDSCs are among them and are a heterogeneous (polymorphonuclear and mononuclear) group of activated, immature myeloid cells and myeloid precursors with potent immune-suppressive activity. All-trans retinoic acid (ATRA) has been shown to negatively affect MDSCs, and it has been used in pre-clinical and clinical studies to inhibit MDSCs by killing polymorphonuclear MDSCs and differentiating mononuclear-MDSCs. Specifically, ATRA treatments substantially (1) decreased the presence of MDSCs in the spleens of tumor-bearing mice and (2) reduced the level of MDSCs in the peripheral blood of patients with renal cell carcinoma, suggesting a role for ATRA in enhancing a potential therapeutic vaccine immune response [25, 26, 29].

On the bases of these observations and findings from a previous trial, we designed a clinical trial to test two worthy hypotheses that could lead to improved efficacy of this therapeutic strategy: (1) chemotherapy may work synergistically with the vaccine’s anti-tumor immune response [22–24] and (2) ATRA administration to decrease the number of MDSCs in patients may improve the vaccine’s anti-tumor immune response [25, 26]. We have previously reported on the results of correlative studies from this trial [27]; here, we report on the participating patients’ clinical outcomes.

## Materials and methods

### Study design

This was a single-institution, randomized phase II study involving patients with extensive-stage SCLC who were responsive to therapy or had non-progressive disease after the first-line conventional chemotherapy. All patients required restaging imaging studies (most often computed tomography imaging of chest or chest and abdomen, with intravenous contrast) following chemotherapy, as is standard practice for this patient population. The decision to designate patients as having “responsive or non-progressive disease” was made clinically by the sub-investigator together with the principal investigator and was based on comparisons of the appropriate radiographic imaging and reports. Brain MRIs were not mandatory screening procedures, but were required for patients with “uncontrolled and/or symptomatic central nervous system metastasis,” which excluded them from the study.

After completion of chemotherapy, patients were randomized in a 1:1:1 fashion to 1 of 3 arms: arm A (control/observation), arm B (Ad.p53-DC vaccine only), or arm C **(**Ad.p53-DC vaccine plus ATRA). At the time of disease progression, all patients were to be treated with the second-line chemotherapy (single-agent paclitaxel; Supplemental Fig. 1) [24, 28].

The primary study endpoint was the overall response rate (ORR) to the second-line paclitaxel. Additional secondary and correlative study endpoints included safety and toxicity of the vaccine, clinical and immunological efficacy of the vaccine, immune correlations to clinical outcomes (median progression-free survival [PFS] and overall survival [OS] based on immune response), and clinical outcomes after the second-line paclitaxel and completion of overall treatment (median PFS and OS). The study was not designed to detect any ORR differences between arms.

### Patient population

Eligible patients were required to have a confirmed pathologic diagnosis of SCLC and extensive-stage disease, good performance status (Eastern Cooperative Oncology Group ≤ 2), age greater than 18 years, normal hematologic, renal, and hepatic function, life expectancy of > 6 months, and measurable disease.

Patients had to have non-progressive disease after 4–6 cycles of chemotherapy with a platinum doublet (etoposide–carboplatin; etoposide–cisplatin), with no other prior therapies. Patients with brain metastases were allowed to enroll if the lesions were treated, asymptomatic, and did not require corticosteroid use. Exclusion criteria included the use of chronic steroids (prednisone > 10 mg); a second malignancy within the past 2 years, except non-melanoma skin cancer or cervical carcinoma in situ; a pre-existing autoimmune disorder or immunodeficiency condition; and women who were pregnant or breastfeeding.

### Vaccine production and release criteria

Eligible patients underwent leukapheresis ≤ 8 weeks after the last dose of chemotherapy to manufacture Ad.p53-DC. The fraction of enriched mononuclear cells recovered from patients was always sufficient to make the vaccine doses (see Supplemental Fig. 2), and the DCs were prepared in our center. The preparation of DCs and their characterization have been previously described [20].

Vaccine-release criteria included (1) negative Gram staining (no organisms seen), (2) negative *mycoplasma* test by polymerase chain reaction analysis, (3) maximum endotoxin concentration of 5 EU/mL, and (4) a mature DC-p53–expressing phenotype. A DC phenotype was defined as cells that were lineage (CD3, CD14, CD19, CD20, and CD56) negative, HLA DR-isotype positive, CD86 positive, and p53 positive. Intracellular p53 staining was performed using the “fix and perm” kit (Caltag, Burlingame, CA) [27].

### Treatments and efficacy assessments

Each vaccine consisted of 2–5 × 10^6^ autologous DCs expressing p53 after infection with adenovirus, encoding the full-length wild-type p53 gene (Ad-p53 DC). Each DC vaccine (1 mL cell suspension) was injected intradermally into four separate sites (0.25 mL at each site), in bilateral proximal upper and lower extremities (regions of the axillary and inguinal nodal basins).

Cells were injected at 2-week intervals three times (three vaccine doses). Patients were monitored for acute toxicity for 1 h after each injection. Patients were restaged approximately 2 weeks after the third vaccine dose. Patients without signs of progressive disease (PD) underwent a second leukapheresis and then received three more vaccines at 4-week intervals.

Patients on arm C also received 150 mg/m^2^ ATRA for 3 days before each vaccine administration (followed by vaccine administration the next day). This scheduling was based on our previous data [26], in which we had observed the persistence of an ATRA effect on MDSCs for a minimum of 2 weeks.

Patients were observed until evidence of disease progression, at which time that they were to receive salvage chemotherapy with single-agent paclitaxel (200 mg/m^2^ on day 1 of a 21-day cycle for 4 cycles). Standard medications to avoid hypersensitivity reactions were administered before paclitaxel; these included 20 mg oral dexamethasone at 6 and 12 h before paclitaxel or 20 mg intravenous dexamethasone plus 50 mg oral diphenhydramine and 50 mg oral ranitidine 30 min before paclitaxel.

For the patients who received vaccines, objective response assessments were performed after the first three vaccine administrations and again after all six were given. Objective responses were also assessed every two cycles during the second-line chemotherapy (paclitaxel). After completion of chemotherapy, follow-up occurred every 3 months.

Response Evaluation Criteria in Solid Tumors (v1.0) were used to assess objective responses and to assess patients during the study. We defined immune responses per Antonia et al. [20].

### Toxicity

Adverse events were collected and graded according to the National Cancer Institute Common Toxicity Criteria for Adverse Events version 4 [29]. Serious adverse events were reported per appropriate safety-reporting requirements.

There were no dose adjustments related to vaccine administration. Patients who did not tolerate ATRA administration were first treated symptomatically (anti-emetics for nausea/vomiting and mild opioids for headaches). If symptoms persisted, ATRA was discontinued in subsequent doses.

For grade 3 or higher toxicity, paclitaxel was withheld until toxicity recovered to ≤ grade 2. The dose of paclitaxel was reduced the first time to 175 mg/m^2^. A second reduction was allowed at the discretion of the treating physician in consultation with the principal investigator. Growth factor support was permitted per American Society of Clinical Oncology guidelines. More than two dose reductions and a delay in paclitaxel administration of more than 3 weeks were not allowed.

### Correlative studies

For the evaluation of immune responses, mononuclear cells were collected from patients at different time points during treatment, following procedures described in Iclozan et al. [27], and kept frozen in liquid nitrogen. All samples from each patient were analyzed simultaneously to reduce inter-experimental variability.

An immune response to vaccine was considered positive if the interferon-γ ELISPOT assay showed ≥ 30 spots per 2 × 10^5^ and the interferon-γ ELISPOT response to a canary pox vector vaccine (ALVAC) p53 was ≥ 2 standard deviations higher than the response to corresponding ALVAC control at the same time point and ≥ 2 standard deviations higher than the corresponding response at baseline.

Cell phenotype analyses and intracellular cytokine staining tests were also performed, following previously reported methodologies [27].

### Statistical analyses

The primary endpoint for the trial was ORR to the second-line paclitaxel. From historical data, we considered an ORR of approximately 25% to the second-line chemotherapy (paclitaxel) as not warranting further study. We were interested in at least 25% (25% versus 50%) improvement in treatment efficacy for arms B and/or C. Therefore, 50% ORR was used as a result to pursue further study.

For each arm, using the two-stage Simon Minimax design [30] with 5% type-I error and 20% type-II error rates, nine patients were enrolled in the first stage of the trial. If two or fewer patients responded, the null hypothesis was rejected and the corresponding treatment arm was stopped. If 3 or more patients showed a response, 15 additional patients (a total of 24 patients per group) were enrolled to the corresponding arm. The study was not designed to detect any ORR differences between arms.

The Wilcoxon–Mann–Whitney test was used to determine associations between groups with continuous variables. Univariate associations between frequencies of responders and groups were analyzed by Fisher’s exact test, due to small sample sizes. Two-sided tests were used for all calculations. *P* < .05 was considered statistically significant.

PFS and OS were calculated by the Kaplan–Meier method [31]. The differences in PFS and OS were examined by the stratified log-rank test. Correlations between the tissue biomarkers and outcomes were computed by the Mantel–Haenszel test. All statistical analyses were conducted using SAS (Statistical Analysis Software) version 9.4 (SAS, Cary, NC).

## Results

The immunological correlative results from our study have been reported previously [27]. Here, we report on the patients’ clinical outcomes (the planned primary endpoint being ORR after paclitaxel and secondary endpoints including PFS and OS) and how they relate to the presence (or absence) of a vaccine-induced immune response.

A factor that influenced our results was that many patients on the trial did not receive salvage chemotherapy with paclitaxel as planned, but received the other types of chemotherapy instead. This happened despite our investigators’ extensive efforts to persuade patients’ chemotherapy providers to treat the on-trial patients in accordance with our protocol.

### Patient characteristics

Between October 2007 and December 2013, 78 patients consented to participate, and 69 patients were ultimately admitted to the trial and randomized; 9 patients were found to be ineligible. Stage 1 of the trial (completed in February 2012) included 55 patients: 18 were enrolled to arm A, 20 to arm B, and 17 to arm C. Patient withdrawal and rapid disease progression prevented patients from completing at least one round of salvage (paclitaxel or other) chemotherapy, creating the need to enroll a larger number of patients than was originally planned.

Arm C met stage 2 criteria for further investigation, resulting in the enrollment of 14 additional patients. Supplemental Fig. 3 (CONSORT [Consolidated Standards of Reporting Trials] diagram) shows the distribution of and treatments received by all 78 patients who consented to participate, and Table 1 shows the characteristics of all 69 patients randomized.

**Table 1:** Patients’ characteristics

Characteristics		Patients, no. (%)			
		Arm A, 18 (26.1)	Arm B, 20 (29.0)	Arm C, 31 (44.9)	Total patients, 69 (100)
Sex	Male	11(15.9)	7 (10.1)	15 (21.7)	33 (47.8)
	Female	7 (10.1)	13 (18.8)	16 (23.2)	36 (52.2)
Age, years	Median	63	63	63	62
	Range	43–73	51–74	48–73	43–74
Race	White	16 (23.2)	19 (27.5)	31 (44.9)	67 (97.1)
	AA/other	1/1 (2.9)	0/0 (0.0)	0/0 (0.0)	1/1 (2.9)
ECOG PS	0	4 (5.8)	6 (8.7)	4 (5.8)	14 (20.3)
	1	14 (20.3)	14 (20.3)	27 (39.1)	55 (79.7)
Chemotherapy	≤ 4 cycles	5 (7.2)	6 (8.7)	4 (5.8)	15 (21.7)
	> 4 cycles	13 (18.8)	14 (20.3)	27 (39.1)	54 (78.3)
Radiotherapy	PCI	5 (7.2)	6 (8.7)	3 (4.3)	14 (20.3)
	WBRT	2 (2.9)	0 (0.0)	7 (10.1)	9 (13.0)
	Thoracic	6 (8.7)	4 (5.8)	4 (5.8)	14 (20.3)
	Distant	2 (2.9)	3 (4.3)	5 (7.2)	10 (14.5)
Leukapheresis	Total (patients)	18 (26.1)	31 (44.9)	49 (71.0)	62 (100)
	Total (quantity)	24 (38.7)	38 (61.3)		
	0/1		2/12 (20.3)	0/24 (34.8)	2/36 (55.1)
	2		6 (8.7)	7 (10.1)	13 (18.8)
Vaccination, No	Median		3	3	3
	Range		0–6	1–6	0–6
	< 3/3/> 3		4/12/4 (29.0)	5/19/7 (44.9)	9/31/11 (73.9)
	Total		59 (36)	105 (64)	164 (100)

*AA* African American, *ECOG PS* Eastern Cooperative Oncology Group performance status, *PCI* prophylactic cranial irradiation, *WBRT* whole-brain radiotherapy

### Safety

As previously described [20, 21], the vaccine was found to be safe with mostly grade 1 or grade 2 toxicities. Only 1 patient receiving vaccine alone experienced grade 3 fatigue, and 8 patients on arm C experienced grade 3 toxicities (one each had fatigue, dyspnea, nausea, vomiting, and mood alteration; three experienced headaches). Five patients in arm C and nine patients in arm B experienced no vaccine-related toxicities (Table 2). Additional toxicities were mostly associated with paclitaxel administration (Supplemental Table 1).

**Table 2:** Vaccine-related toxicities included in the standard abbreviations

	Ad.p53-DC vaccines + ATRA (*n* = 31)				Ad.p53-DC vaccines (*n* = 20)				Total (*N* = 51)	
	*G1*/*G2*	*G3*	Total	%	*G1*/*G2*	*G3*	Total	%		**%**
*Laboratory, metabolic*/*chemistry*										
Sodium, serum-low (hyponatremia)	2		2	6.45				0.00	2	3.92
*Laboratory, hematologic*										
Hemoglobin	1		1	3.23	1		1	5.00	2	3.92
Neutrophils/granulocytes	1		1	3.23	1		1	5.00	2	3.92
Platelets	2		2	6.45				0.00	2	3.92
*Constitutional*										
Anorexia	5		5	16.13	2		2	10.00	7	13.73
Fatigue (asthenia, lethargy, and malaise)	9	1	10	32.26	2	1	3	15.00	13	25.49
Edema (limb, head, and neck)	1		1	3.23				0.00	1	1.96
*Respiratory*										
Cough	3		3	9.68	1		1	5.00	4	7.84
Dyspnea (shortness of breath)	5	1	6	19.35	2		2	10.00	8	15.69
Hypoxia				0.00	1		1	5.00	1	1.96
*Gastrointestinal*										
Constipation	3		3	9.68	1		1	5.00	4	7.84
Diarrhea	6		6	19.35				0.00	6	11.76
Nausea	8	1	9	29.03	1		1	5.00	10	19.61
Vomiting	6	1	7	22.58				0.00	7	13.73
*Neurologic*/*psychiatric*										
Pain/headache	19	3	22	70.97				0.00	22	43.14
Neuropathy (sensory/motor)	1		1	3.23	3		3	15.00	4	7.84
Mood alterations (agitation, anxiety, depression, and euphoria)		1	1	3.23	1		1	5.00	2	3.92
*Cutaneous*										
Injection site reaction/extravasation changes	2		2	6.45	3		3	15.00	5	9.80
Rash (desquamation, acneiform, treatment associated, and hay fever reaction)	2		2	6.45				0.00	2	3.92
Dry skin	4		4	12.90				0.00	4	7.84
Pruritus/itching	2		2	6.45				0.00	2	3.92
Hair loss/alopecia (scalp or body)	2		2	6.45				0.00	2	3.92
*Musculoskeletal*										
Pain (arthralgia/myalgia)	3		3	9.68				0.00	3	5.88
*Pain*										
Abdominal and not otherwise specified	1		1	3.23				0.00	1	1.96
Back and neck	1		1	3.23	1		1	5.00	2	3.92
Extremities and limbs	2		2	6.45	1		1	5.00	3	5.88
Other	2		2	6.45				0.00	2	3.92
*Miscellaneous*										
Urinary retention (including neurogenic bladder)	1		1	3.23				0.00	1	1.96

Ad.p53-DC, *TP53* transfected dendritic cell-based vaccine, *ATRA* all-trans-retinoic acid, *DC* dendritic cells, *G* grade

### Immune response analyses

**Fig. 1:**
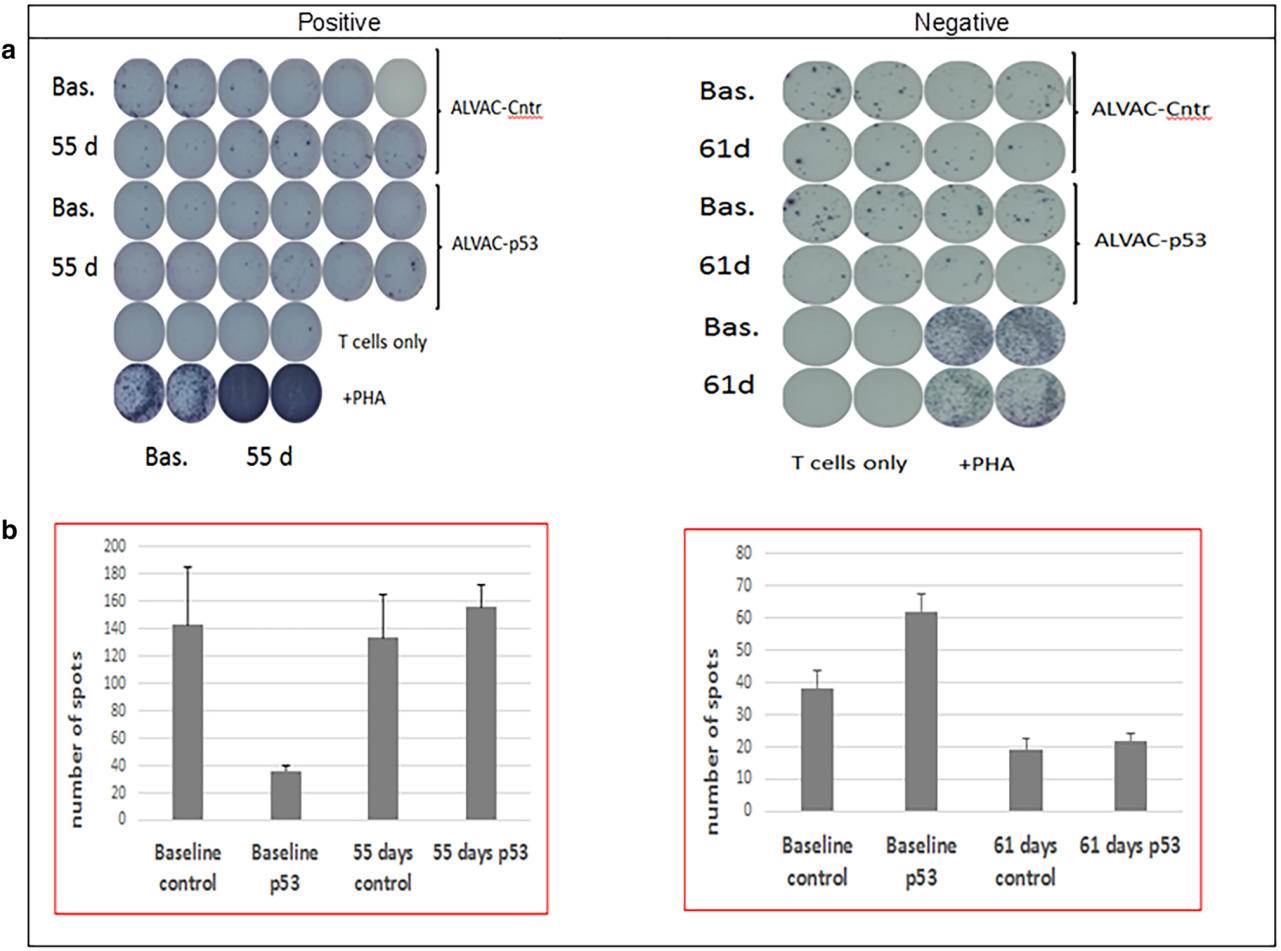
Positive and negative interferon-γ ELISPOT assay results (examples from 2 arm-C patients). **a** PBMCs were seeded in 96-well plates that were precoated with an anti–interferon-γ antibody. T-cell responses were assessed after the addition of a recombinant canarypox virus (ALVAC) containing wild-type p53 or empty vector (empty ALVAC virus served as a control). Additional controls were prepared (unstimulated or phytohemagglutinin-stimulated cells). **b** Number of interferon-γ–producing cells was evaluated (counted) using an automated ELISPOT reader. Abbreviations: ALVAC, canary pox vector vaccine; Bas, baseline; cntr, control; d, day; PHA, phytohemagglutinin

Paired serum samples were tested for immune responses, using an interferon-γ ELISPOT assay and following previously described methodology [27], and were available in 5/18 patients in arm A, 15/20 patients in arm B, and 23/31 patients in arm C. No immune responses were detected in arm-A patients, and positive immune responses were obtained in 3 arm-B patients (3/15, 20.0%; 95% confidence interval [CI], 5.3–48.6%) and in 10 arm C patients (10/23, 43.3%; 95% CI 23.9–65.1%). Figure 1 shows negative and positive results for two of the patients enrolled in arm C.

### Clinical efficacy

At stage 1, 20 patients died before receiving salvage chemotherapy (5, 7, and 8 patients in arms A, B, and C, respectively). One other patient in arm C died before salvage chemotherapy during stage 2. Most of these patients (3, 3, and 4 in arms A, B, and C, respectively) developed brain metastases and did not recover sufficiently to be able to receive an additional therapy before death.

The large patient dropout rate was due in part due to a flawed trial design that introduced a significant bias to our results. As mentioned, a number of the patients who received salvage chemotherapy outside our center received agents other than paclitaxel. In an attempt to minimize the effects of the introduced bias, we chose to report on the objective response achieved to any second-line chemotherapy received at the time of PD.

#### Objective response to paclitaxel or other second-line chemotherapy

**Fig. 2:**
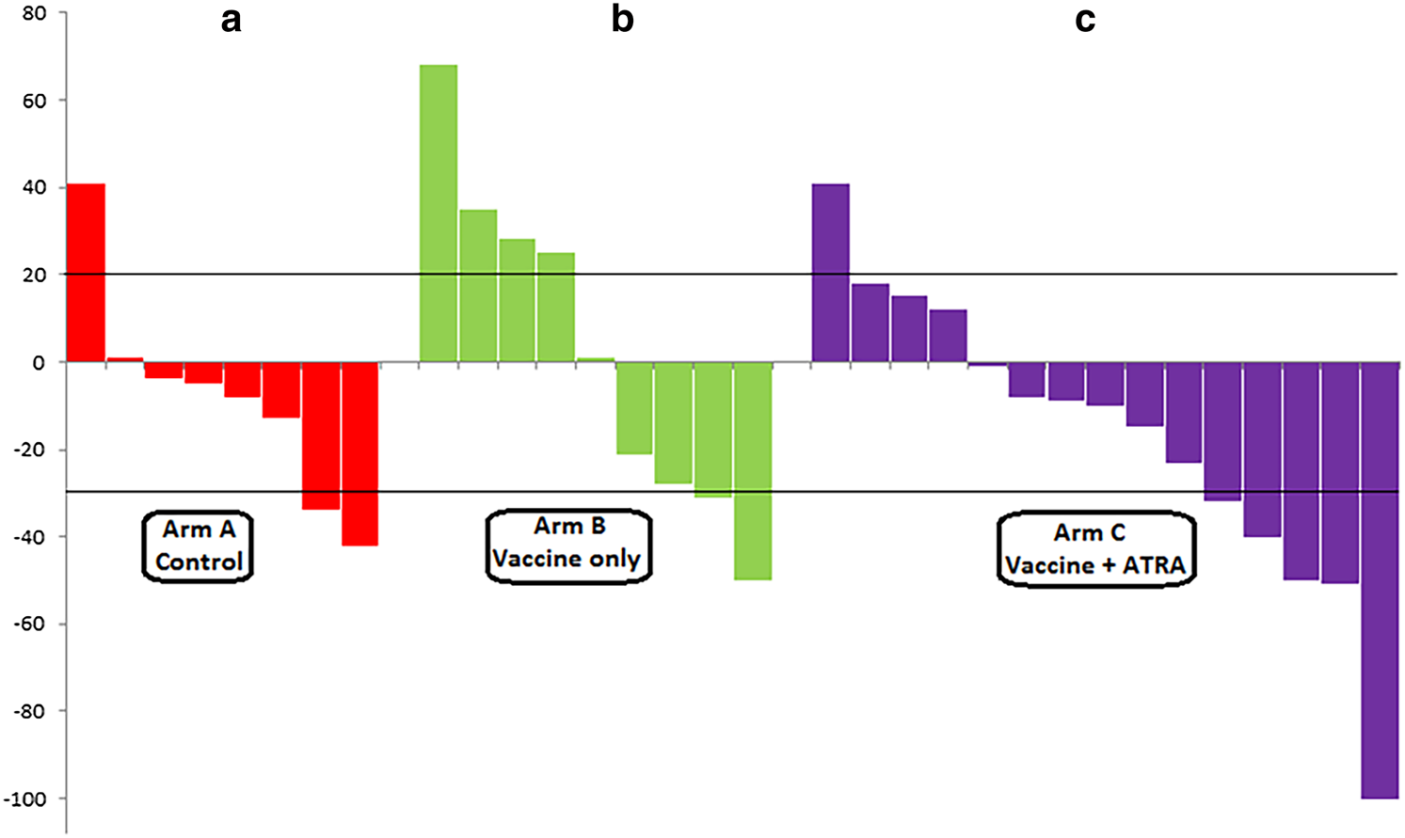
Clinical response to salvage chemotherapy (paclitaxel and others) by patient (waterfall plot). Response was defined per Response Evaluation Criteria in Solid Tumors (%). **a** Control or observation (no vaccine), **b** Vaccine only, **c** Vaccine plus all-trans-retinoic acid (ATRA)

In arm A, 7 patients received non-paclitaxel treatment (4 had etoposide-carboplatin, 1 had topotecan, 1 had cyclophosphamide-doxorubicin-vincristine, and 1 was treated on a different clinical trial), and 6 received paclitaxel. The ORR could only be verified for 9 patients, which included 2 with PRs, 6 with stable disease (SD), and 1 with PD (2/13, 15.4%; 95% CI 2.7–46.3%) (Fig. 2a). In arm B, 1 patient did not progress, 1 received non-paclitaxel chemotherapy (irinotecan–platinum instead), and 11 patients received paclitaxel. ORR could only be verified in 11 patients: 2 with PRs, 3 with SD, and 6 with PD (2/12, 16.7%; 95% CI 2.9–49.1%) (Fig. 2b). In the arm-C group at stage 1 (*n* = 17), 1 patient did not progress, 1 received non-paclitaxel chemotherapy (topotecan), and 7 received paclitaxel. Overall responses could only be verified for 6 of these patients: 1 with complete response, 2 with PRs, 2 with SD, and 2 with PD (3/8, 37.5%; 95% CI 10.2–74.1%). In arm C, at stage 2 (*n* = 14), 2 patients received non-paclitaxel chemotherapy (clinical trials) and 11 patients received paclitaxel. Overall responses were verified in all of these patients: 2 had PRs, 5 had SD, and 6 had PD (2/13, 15.4%; 95% CI 2.7–46.3%). The ORR for all arm-C patients was 23.8% (5/21 patients; 95% CI 9.1–47.5%) (Fig. 2c). Although our study was not designed to detect differences in the ORR, Supplemental Table 2 shows the ORRs for each arm and a calculation of the differences in stage 1 response rates using Fisher’s exact test. There were no statistically significant differences.

#### Objective response to vaccination

**Fig. 3:**
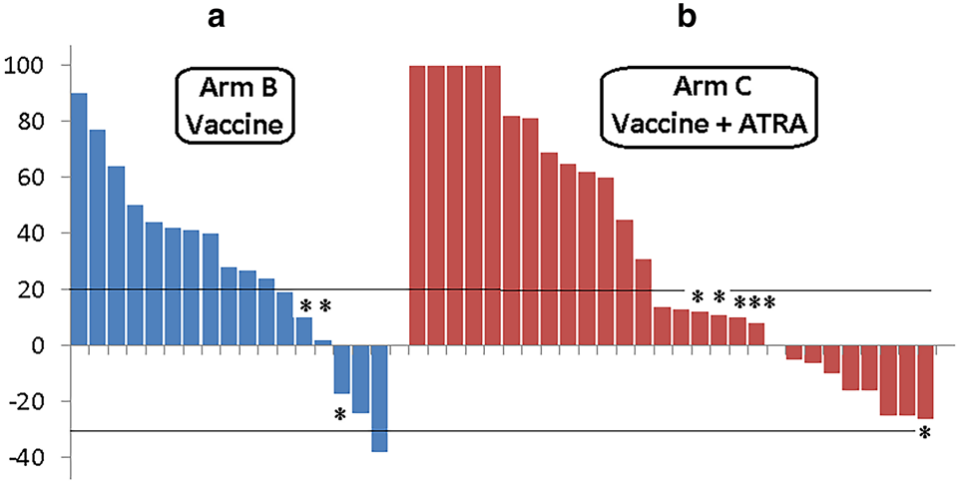
Clinical response to vaccine by patient (waterfall plot). Response was defined per response evaluation criteria in solid tumors (%). **a** Vaccine only; **b** vaccine plus all-trans-retinoic acid (ATRA). *Patients with new central nervous system disease (brain metastasis)

In arm B, 2 patients maintained their pre-vaccination complete response (no measurable disease) and 1 patient chose not to return for additional vaccinations after a post-vaccination positron emission tomography scan documented SD. Among the other 17 patients, 1 showed a PR, 3 had SD, and 13 had PD (3 patients developed new lesions despite SD after vaccination) (Fig. 3a). In arm C, 2 patients had clear clinical PD, while, on vaccine treatment, and a third had an outside scan report that also confirmed PD. Of the remaining 28 patients, 9 had SD and 19 had PD (6 patients developed new lesions despite SD after vaccination) (Fig. 3b). Thus, we observed an objective response to vaccination of 1/45 (2.2%; 95% CI 0.1–13.2%) and stabilization of disease in an additional 12/45 patients (26.7%; 95% CI 15.1–42.2%).

#### Survival

**Fig. 4:**
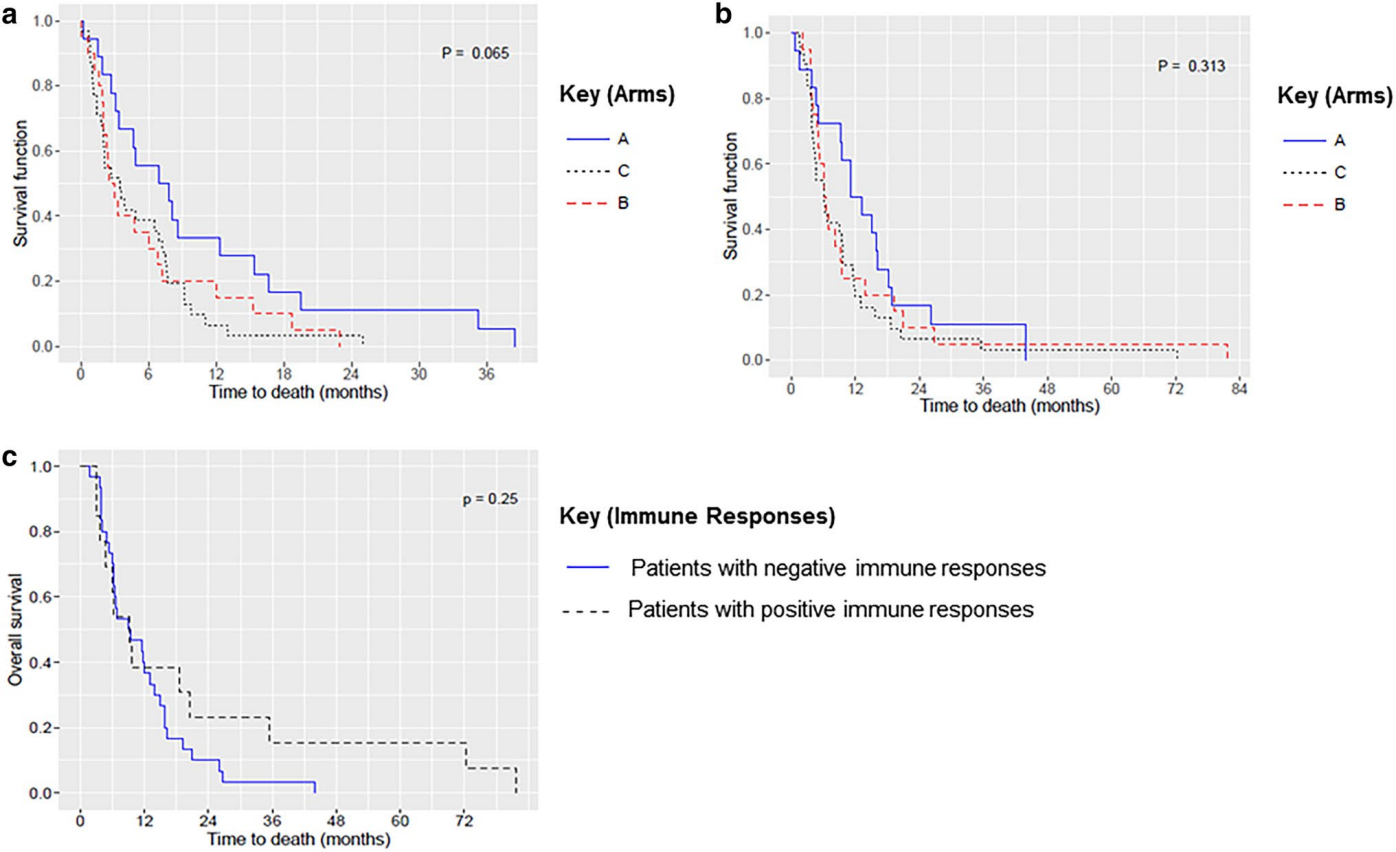
Kaplan–Meier overall survival curves. **a** Between-arm comparison, from the date of progression after vaccination or, for those in the control arm, after enrollment, **b** between-arm comparison from the date of study enrollment, and **c** immune response comparison, with survival calculated on the basis of the immune response achieved by vaccination (positive or negative)

OS in each arm was analyzed in two different ways: (1) from the date of progression after vaccination for those in arms B and C or the date of progression after enrollment for those in the control arm (arm A) (Fig. 4a) and (2) from the date of study enrollment (Fig. 4b). Although no statistically significant differences were found, OS in arm A was consistently numerically superior in both scenarios (Supplemental Table 3). OS was also analyzed based on the immune response achieved by vaccination (Fig. 4c). No statistically significant difference was seen between patients with positive and negative immune responses (9.2 versus 9.3 months, respectively; *P* = .250) (Supplemental Table 2).

## Discussion

To test our hypothesis (vaccine immunization enhances response to chemotherapy) [20, 21], we designed a randomized, controlled phase II study, in which the primary endpoint assessment (response to chemotherapy after vaccination) was at the backend of the design (Supplemental Fig. 1). This design proved to be flawed due to the large number of patients who dropped out or came off study (because of death, clinical deterioration precluding further treatment, choice of having chemotherapy at home, being lost to follow-up, and withdrawal of consent after randomization to control arm) before their results could be evaluated.

Although the flawed design extended the first stage of our trial by increasing the number of patients required to enroll to reach the desired evaluable number of patients (which increased the potential for additional selection bias to have been introduced), it did not impede the collection and analysis of the results for our primary endpoint. Unfortunately, despite arm C meeting criteria for expansion to the second stage of the trial, our study failed to reach the desired final endpoint, as the final ORR to salvage chemotherapy in arm C was only 23.8% (95% CI 9.1–47.5%).

It was also surprising and especially disappointing that the secondary outcome of OS in our experimental arms (arms B and C) fell short of being better than it was in the control/observation arm (A). In fact, although not statistically significant, the median survival time (MST) for the control arm was numerically superior to both experimental arms (Supplemental Table 3). The reasons for these findings are unclear, but the flawed trial design and the potential selection bias introduced in a relatively small population (that is, small sample size) should be of particular consideration. The potential influence of selection bias is highlighted by the fact that the control arm in our study also appeared to outperform the results (MST) from the other previously reported trials and treatment regimens [32].

Despite these limitations, our trial allowed us to conclude that (1) the Ad.p53-DC vaccine is safe and elicits a specific cytotoxic T-cell immune response in 20–40% of patients; (2) an ORR to the second-line chemotherapy of 24% in the setting of recurrent SCLC is an acceptable rate that favorably compares with topotecan, the only currently approved agent for this indication [33–35]; (3) although MST was not different and acknowledging the small sample size, immune response-positive patients seem to have had a “late stabilization/flattening” of the survival curve, and three long-term survivors (> 5 years) were observed among these patients, including two who never progressed, never received the second-line chemotherapy, and died from other causes (not SCLC), and all patients with a negative immune response died from PD; (4) manipulation of the tumor microenvironment (cancer-immunity cycle [36–39]) by reducing the number of immunosuppressive MDSCs with ATRA improved the specific cytotoxic immune response (data presented previously [27]).

These findings (particularly the fourth) suggest that further evaluation of this Ad.p53-DC vaccine and the role that it may play in the treatment of SCLC (and potentially other p53-overexpressing tumors) should be considered in the context of combinations with the other immunomodulatory drugs in an attempt to better remove/reduce the immunosuppressive effects in the tumor microenvironment and create a more susceptible milieu for immunization, which should yield a positive immunotherapeutic anti-tumor response.

Certainly, the evidence accumulated recently with the newer anti–PD-1 and/or anti–CTLA-4 checkpoint inhibitors, either as monotherapies (such as nivolumab [40] or pembrolizumab [41]) or in combination treatments (such as ipilimumab plus nivolumab) [40]), suggests these as the leading compounds for future combinatorial investigations. However, many other agents, with different molecular structures, pharmacokinetic and pharmacodynamic properties, biologic activities, and mechanisms of action (sunitinib malate [42], 1-methyl-DL-tryptophan [43], and anti-CD40 [44] monoclonal antibodies) but similar immunostimulatory activity, exist that are under clinical investigation.

## Conclusions

Our vaccine failed to improve the response rate to paclitaxel. However, its encouraging safety profile and therapeutic immune potential remain in place. The consideration of further research combining Ad.p53 DC vaccine with different immunostimulatory agents is reasonable and warranted for the pursuit of better therapeutic options for SCLC patients. Indeed, we have a trial in a relapsed/recurrent (platinum chemotherapy failure) SCLC population under way, in which Ad.p53-DC is combined with ipilimumab plus nivolumab (NCT03406715). Building on our learning from the two prior trials with Ad.p53-DC, the current investigational protocol restricts accrual to those patients with p53 protein tissue expression above 50% and incorporates the measurement of tumor mutation burden, in an attempt to develop predictive biomarkers for this combinatorial immunotherapeutic strategy.

## Electronic supplementary material

Below is the link to the electronic supplementary material.

Supplementary material 1 (PDF 231 KB)

## Abbreviations

ATRA: All-trans-retinoic acid
CI: Confidence interval
DC: Dendritic cell
MDSC: Myeloid-derived suppressor cell
ORR: Overall response rate
OS: Overall survival
PD: Progressive disease
PFS: Progression-free survival
PR: Partial response
SCLC: Small cell lung cancer
SD: Stable disease

## References

[1] Bouaoun L, Sonkin D, Ardin M, Hollstein M, Byrnes G, Zavadil J, Olivier M (2016). TP53 Variations in human cancers: new lessons from the IARC TP53 database and genomics data. Hum Mutat.

[2] Hollstein M, Sidransky D, Vogelstein B, Harris CC (1991). p53 mutations in human cancers. Science.

[3] Wistuba II, Gazdar AF, Minna JD (2001). Molecular genetics of small cell lung carcinoma. Semin Oncol.

[4] Davies AM, Lara PN, Lau DH, Gandara DR (2004). Treatment of extensive small cell lung cancer. Hematol Oncol Clin North Am.

[5] Ettinger DS (2001). New drugs for chemotherapy-naive patients with extensive-disease small cell lung cancer. Semin Oncol.

[6] Hanna N, Bunn PA, Langer C (2006). Randomized phase III trial comparing irinotecan/cisplatin with etoposide/cisplatin in patients with previously untreated extensive-stage disease small-cell lung cancer. J Clin Oncol.

[7] Johnson BE (2002). Management of small cell lung cancer. Clin Chest Med.

[8] Johnson DH (1999). Management of small cell lung cancer: current state of the art. Chest.

[9] Lara PN, Gandara DR, Natale RB (2006). Randomized phase III trial of cisplatin/irinotecan versus cisplatin/etoposide in patients with extensive-stage small-cell lung cancer. Clin Lung Cancer.

[10] Simon GR, Wagner H (2003). Small cell lung cancer. Chest.

[11] Socinski MA, Smit EF, Lorigan P (2009). Phase III study of pemetrexed plus carboplatin compared with etoposide plus carboplatin in chemotherapy-naive patients with extensive-stage small-cell lung cancer. J Clin Oncol.

[12] Vierboom MP, Nijman HW, Offringa R (1997). Tumor eradication by wild-type p53-specific cytotoxic T lymphocytes. J Exp Med.

[13] Zwaveling S, Vierboom MP, Ferreira Mota SC (2002). Antitumor efficacy of wild-type p53-specific CD4(+) T-helper cells. Cancer Res.

[14] George J, Lim JS, Jang SJ (2015). Comprehensive genomic profiles of small cell lung cancer. Nature.

[15] Lohmann D, Putz B, Reich U, Bohm J, Prauer H, Hofler H (1993). Mutational spectrum of the p53 gene in human small-cell lung cancer and relationship to clinicopathological data. Am J Pathol.

[16] Peifer M, Fernandez-Cuesta L, Sos ML (2012). Integrative genome analyses identify key somatic driver mutations of small-cell lung cancer. Nat Genet.

[17] Nikitina EY, Chada S, Muro-Cacho C, Fang B, Zhang R, Roth JA, Gabrilovich DI (2002). An effective immunization and cancer treatment with activated dendritic cells transduced with full-length wild-type p53. Gene Ther.

[18] Nikitina EY, Clark JI, Van Beynen J, Chada S, Virmani AK, Carbone DP, Gabrilovich DI (2001). Dendritic cells transduced with full-length wild-type p53 generate antitumor cytotoxic T lymphocytes from peripheral blood of cancer patients. Clin Cancer Res.

[19] Ishida T, Chada S, Stipanov M, Nadaf S, Ciernik FI, Gabrilovich DI, Carbone DP (1999). Dendritic cells transduced with wild-type p53 gene elicit potent anti-tumour immune responses. Clin Exp Immunol.

[20] Antonia SJ, Mirza N, Fricke I (2006). Combination of p53 cancer vaccine with chemotherapy in patients with extensive stage small cell lung cancer. Clin Cancer Res.

[21] Chiappori AA, Soliman H, Janssen WE, Antonia SJ, Gabrilovich DI (2010). INGN-225: a dendritic cell-based p53 vaccine (Ad.p53-DC) in small cell lung cancer: observed association between immune response and enhanced chemotherapy effect. Expert Opin Biol Ther.

[22] Schlom J, Arlen PM, Gulley JL (2007). Cancer vaccines: moving beyond current paradigms. Clin Cancer Res.

[23] Wheeler CJ, Das A, Liu G, Yu JS, Black KL (2004). Clinical responsiveness of glioblastoma multiforme to chemotherapy after vaccination. Clin Cancer Res.

[24] Ramakrishnan R, Antonia S, Gabrilovich DI (2008). Combined modality immunotherapy and chemotherapy: a new perspective. Cancer Immunol Immunother.

[25] Kusmartsev S, Cheng F, Yu B, Nefedova Y, Sotomayor E, Lush R, Gabrilovich D (2003). All-trans-retinoic acid eliminates immature myeloid cells from tumor-bearing mice and improves the effect of vaccination. Cancer Res.

[26] Mirza N, Fishman M, Fricke I, Dunn M, Neuger AM, Frost TJ, Lush RM, Antonia S, Gabrilovich DI (2006). All-trans-retinoic acid improves differentiation of myeloid cells and immune response in cancer patients. Cancer Res.

[27] Iclozan C, Antonia S, Chiappori A, Chen DT, Gabrilovich D (2013). Therapeutic regulation of myeloid-derived suppressor cells and immune response to cancer vaccine in patients with extensive stage small cell lung cancer. Cancer Immunol Immunother.

[28] Ettinger DS (1996). Single-agent paclitaxel in the treatment of small cell lung cancer. Semin Oncol.

[29] Bunn PA, Carney DN (1997). Overview of chemotherapy for small cell lung cancer. Semin Oncol.

[30] Simon R (1989). Optimal two-stage designs for phase II clinical trials. Control Clin Trials.

[31] Kaplan EL, Meier P (1958). Nonparametric estimation from incomplete observations. J Am Stat Assoc.

[32] Owonikoko TK, Behera M, Chen Z, Bhimani C, Curran WJ, Khuri FR, Ramalingam SS (2012). A systematic analysis of efficacy of second-line chemotherapy in sensitive and refractory small-cell lung cancer. J Thorac Oncol.

[33] O’Brien ME, Ciuleanu TE, Tsekov H (2006). Phase III trial comparing supportive care alone with supportive care with oral topotecan in patients with relapsed small-cell lung cancer. J Clin Oncol.

[34] von Pawel J, Schiller JH, Shepherd FA (1999). Topotecan versus cyclophosphamide, doxorubicin, and vincristine for the treatment of recurrent small-cell lung cancer. J Clin Oncol.

[35] von Pawel J, Jotte R, Spigel DR (2014). Randomized phase III trial of amrubicin versus topotecan as second-line treatment for patients with small-cell lung cancer. J Clin Oncol.

[36] Chen DS, Mellman I (2013). Oncology meets immunology: the cancer-immunity cycle. Immunity.

[37] Chen DS, Mellman I (2017). Elements of cancer immunity and the cancer-immune set point. Nature.

[38] Herbst RS, Soria JC, Kowanetz M (2014). Predictive correlates of response to the anti-PD-L1 antibody MPDL3280A in cancer patients. Nature.

[39] Mellman I, Hubbard-Lucey VM, Tontonoz MJ (2016). De-risking immunotherapy: report of a consensus workshop of the cancer immunotherapy consortium of the Cancer Research Institute. Cancer Immunol Res.

[40] Antonia SJ, Lopez-Martin JA, Bendell J (2016). Nivolumab alone and nivolumab plus ipilimumab in recurrent small-cell lung cancer (CheckMate 032): a multicentre, open-label, phase 1/2 trial. Lancet Oncol.

[41] Ott PA, Elez E, Hiret S, Kim DW, Morosky A, Saraf S, Piperdi B, Mehnert JM (2017). Pembrolizumab in patients with extensive-stage small-cell lung cancer: results from the phase Ib KEYNOTE-028 study. J Clin Oncol.

[42] Ozao-Choy J, Ma G, Kao J (2009). The novel role of tyrosine kinase inhibitor in the reversal of immune suppression and modulation of tumor microenvironment for immune-based cancer therapies. Cancer Res.

[43] Cady SG, Sono M (1991). 1-Methyl-DL-tryptophan, beta-(3-benzofuranyl)-DL-alanine (the oxygen analog of tryptophan), and beta-[3-benzo(b)thienyl]-DL-alanine (the sulfur analog of tryptophan) are competitive inhibitors for indoleamine 2,3-dioxygenase. Arch Biochem Biophys.

[44] Vonderheide RH, Flaherty KT, Khalil M (2007). Clinical activity and immune modulation in cancer patients treated with CP-870,893, a novel CD40 agonist monoclonal antibody. J Clin Oncol.

