# Supplementary material for: Randomized-controlled phase II trial of salvage chemotherapy after immunization with a TP53-transfected dendritic cell-based vaccine (Ad.p53-DC) in patients with recurrent small cell lung cancer
Source: Cancer Immunol Immunother. 2018 Dec 27;68(3):517–27. doi: 10.1007/s00262-018-2287-9 (PMC6426813; doi:10.1007/s00262-018-2287-9)
Supplement: Supplementary file 1 — Supplementary material 1 (PDF 231 KB) [file 262_2018_2287_MOESM1_ESM.pdf]

Supplemental Figure 1

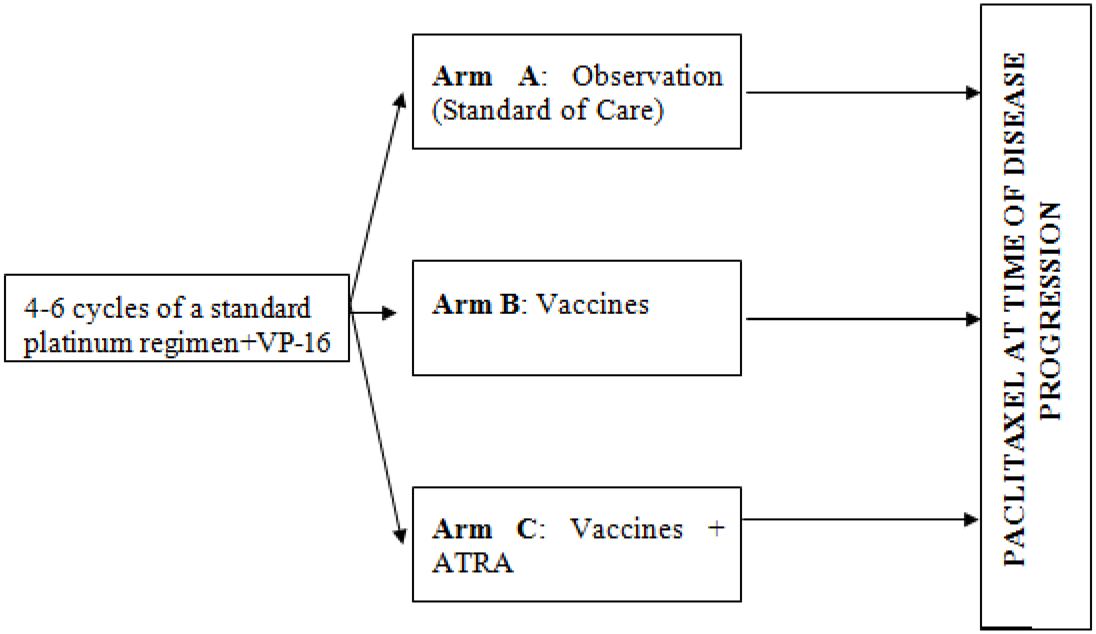

Study design diagram showing treatment received by patients in each study arm. Abbreviations: ATRA, all-trans-retinoic acid; VP-16, etoposide.

Supplemental Figure 2

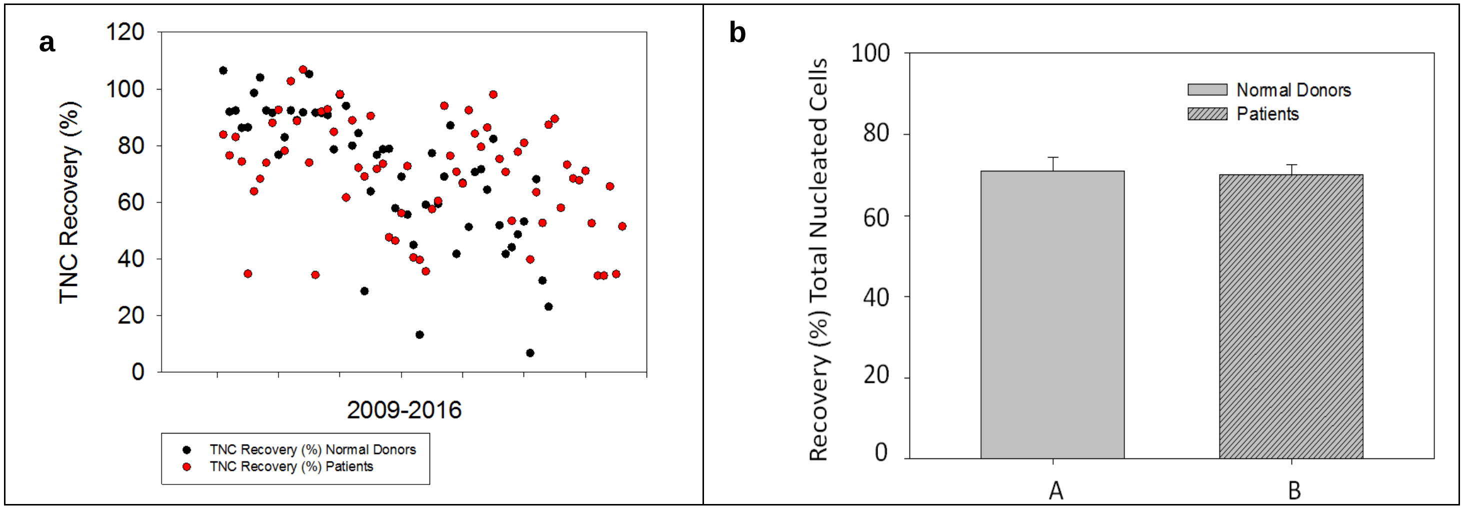

Total nucleated cell (TNC) recovery percentages. **a** Black dots represent normal donors (n = 54) and red dots represent patients (n = 66) who underwent a standard 4-blood-volume apheresis procedure. **b** Graph shows percentages of TNCs for normal donors (A) and patients (B). The apheresis products were subjected to ficoll separation using a Haemonetics Cell Saver 5+ automated cell recovery system to prepare an enriched mononuclear cell preparation. Data are expressed as means ± SEMs percent recovery of total nucleated cells, following ficoll separation of apheresis products from normal donors versus patients (71 ± 3% versus 70 ± 2%, *P* = .511, respectively).

Supplemental Figure 3

CONSORT Flow Diagram

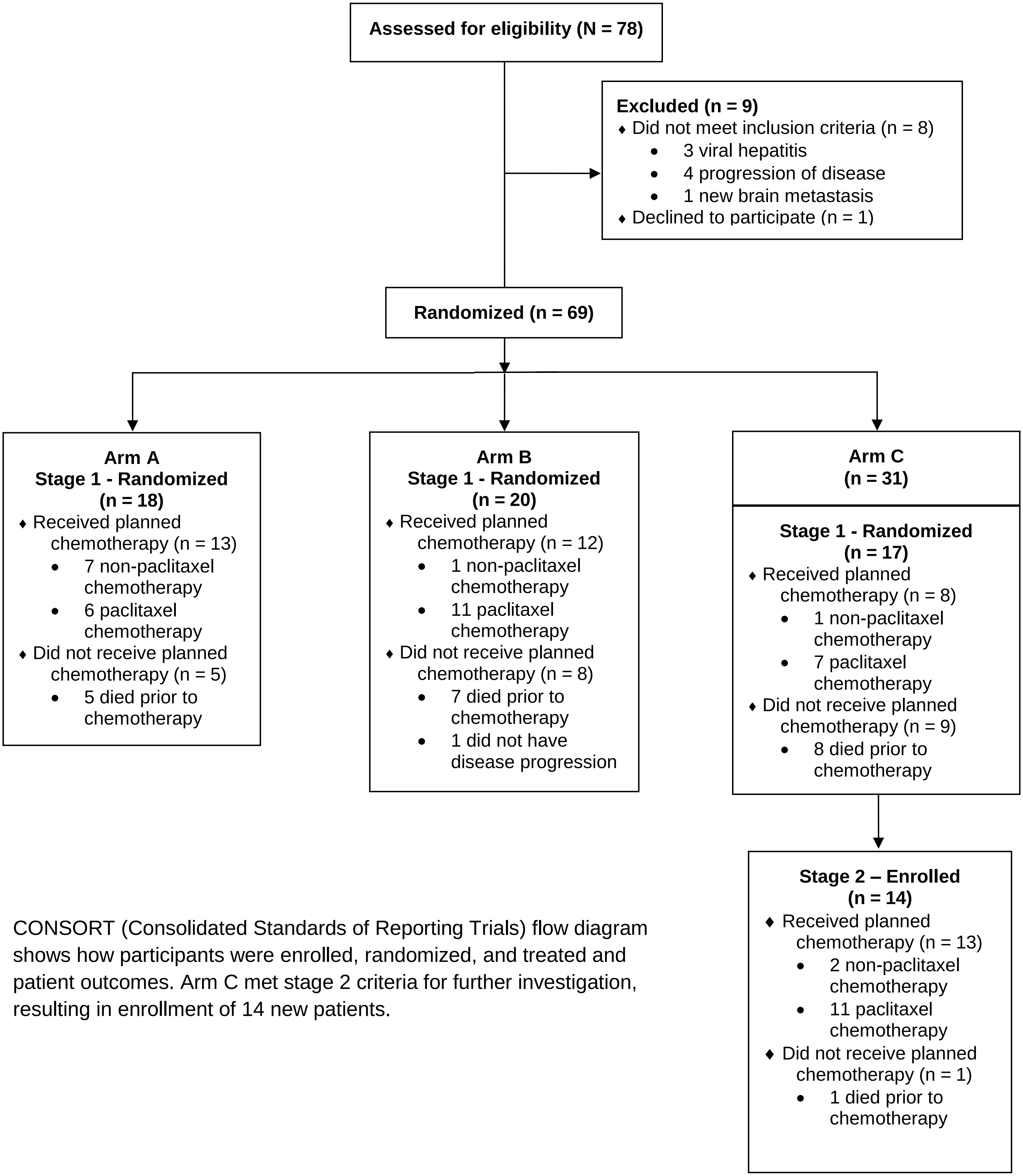

CONSORT (Consolidated Standards of Reporting Trials) flow diagram shows how participants were enrolled, randomized, and treated and patient outcomes. Arm C met stage 2 criteria for further investigation, resulting in enrollment of 14 new patients.

Supplemental Table 1 Adverse events by study arm

Adverse Events	Arm A						Arm B						Arm C						Totals (Arms A, B, and C)					
	N = 18*			n = 16**			N = 20			n = 17			N = 31			n = 31			N = 69			n = 64		
	G1	G2	G3	G4	G3+4 (%)	Total, No. (%)	G1	G2	G3	G4	G3+4 (%)	Total, No. (%)	G1	G2	G3	G4	G3+4 (%)	Total, No. (%)	G1	G2	G3	G4	G3+4 (%)	Total, No. (%)
**Laboratory-Metabolic/Chemistries**																								
Alkaline phosphatase	1		1		5.6	2 (11.1)					0		3	1			0	4 (12.9)	4	1	1		1.4	6 (8.7)
**Laboratory-Hematologic**																								
Lymphopenia			1		5.6	1 (5.6)					0		1	1		1	3.2	3 (9.7)	1	1	1	1	2.9	4 (5.8)
Platelets	1				0	1 (5.6)	3				0	3 (15.0)	4		3		9.7	7 (22.6)	8		3		4.3	11 (15.9)
**Constitutional**																								
Anorexia	4	2	1		5.6	7 (38.9)	5				0	5 (25.0)	10	2			0	12 (38.7)	19	4	1		1.4	24 (34.8)
Fatigue (asthenia, lethargy, malaise)	5	2	2		11.1	9 (50.0)	9	3	1		5.0	13 (65.0)	14	6	2		6.5	22 (71.0)	28	11	5		7.2	44 (63.8)
Fever†	1		1		5.6	2 (11.1)	1				0	1 (5.0)	1				0	1 (3.2)	3		1		1.4	4 (5.8)
**Respiratory**																								
Dyspnea	5	2	2		11.1	9 (50.0)	4	6	1		5.0	11 (55.0)	7	7	1		3.2	15 (48.4)	16	15	4		5.8	35 (50.7)
**Gastrointestinal**																								
Vomiting			1		5.6	1 (5.6)	1	1			0	2 (10.0)	6	2	1		3.2	9 (29.0)	7	3	2		2.9	12 (17.4)
**Neurologic and Psychiatric**																								
Pain- Headache	3		1		5.6	4 (22.2)	3		1		5.0	4 (20.0)	19	2	4		12.9	25 (80.6)	25	2	6		8.7	33 (47.8)
Dizziness	3		1		5.6	4 (22.2)	1				0	1 (5.0)	2	1			0	3 (9.7)	6	1	1		1.4	8 (11.6)
Confusion			1		5.6	1 (5.6)		1			0	1 (5.0)					0			1	1		1.4	2 (2.9)
Seizure		1	1		5.6	2 (11.1)					0						0			1	1		1.4	2 (2.9)
**Cutaneous**																								
Alopecia (scalp or body)		3			0	3 (16.7)	1	1	1		5.0	3 (15.0)	2	6			0	8 (25.8)	3	10	1		1.4	14 (20.3)
**Pain**																								
Pain- Extremity/limb	1	2			0	3 (16.7)	3				0	3 (15.0)		4	1	1	6.5	6 (19.4)	4	6	1	1	2.9	12 (17.4)
Pain- Bone					0								4	1	2		6.5	7 (22.6)	4	1	2		2.9	7 (10.1)

*N = no. of patients in arm; **n = no. of patients who experienced adverse events; †Fever defined in the absence of neutropenia, where neutropenia is defined as absolute neutrophil count < 1.0 x 10e9/L. Abbreviation: G, grade.

Supplemental Table 2 Patient overall response rates to second-line chemotherapy

Arm, Stage	Randomize/ Enrolled, n	Did Not Receive Salvage Chemo Tx, no.			Tx with Salvage Chemo, no.											ORR #1, n/N*, % (range, 95% CI)	ORR #2, n/N**, % (range, 95% CI)	ORR #3, n/N†, % (range, 95% CI)	ORR #4, n/N‡, % (range, 95% CI)
					Paclitaxel					Non-paclitaxel					Total				
		Died	Survived	Total Treated	PR	SD	PD	UnV	Sub-total	PR	SD	PD	UnV	Sub-total					
A, 1	18	5	0	5	2	4	0	0	6	0	2	1	4	7	13	2/18, 11.1 (1.9, 36.1)	2/13, 15.4 (2.7, 46.3)	2/6, 33.3 (6.0, 75.9)	0/7, 0.0 (0.0, 43.9)
B, 1	20	7	1	8	1	3	6	1	11	1	0	0	0	1	12	2/20, 10.0 (1.8, 33.1)	2/12, 16.7 (2.9, 49.1)	2/11, 18.2 (3.2, 52.2)	1/1, 100 (5.5, 100.0)
C, 1 & 2	31	9	1	10	5	5	8	0	18	0	2	0	1	3	21	5/31, 16.1 (6.1, 34.5)	5/21, 23.8 (9.1, 47.5)	5/18, 27.8 (10.7, 53.6)	0/3, 0.0 (0.0, 69.0)
C, 1	17	8	1	9	3	2	2	0	7	0	0	0	1	1	8	3/17, 17.6 (4.7, 44.2)	3/8, 37.5 (10.2, 74.1)	3/7, 42.9 (11.8, 79.8)	0/1, 0.0 (0.0, 94.5)
C, 2	14	1	0	1	2	3	6	0	11	0	2	0	0	2	13	2/14, 14.3 (2.5, 43.8)	2/13, 15.4 (2.7, 46.3)	2/11, 18.2 (3.2, 52.2)	0/2, 0.0 (0.0, 80.2)
All (Totals)	69	21	2	23	8	12	14	1	35	1	4	1	5	11	46	9/69, 13.0 (6.5, 23.8)	9/46, 19.6 (9.9, 34.4)	8/35, 22.9 (11.0, 40.6)	1/11, 9.1 (0.5, 42.9)
¶*P* values for **stage 1 responses only,** calculated using Fisher exact test																.779	.485	.529	.222

Abbreviations: ORR, overall response rate; PD, progressive disease; PR, partial response; SD, stable disease; Tx, treatment; UnV, unevaluable.
*Response/all randomized; **Response/treated only; †Response/paclitaxel-treated only; ‡Response/non-paclitaxel-treated only.

Overall response rates (ORR) to second-line chemotherapy. Arm C met stage 2 criteria for further investigation, and 14 new patients were enrolled in Arm C, Stage 2. ORR #1 is the response rate for all patients who were randomized to the trial. ORR#2 is the response rate for treated patients only. ORR #3 is the response rate for paclitaxel-treated patients. ORR #4 is the response rated for patients who were treated with a second-line chemotherapy other than paclitaxel.

Supplemental Table 3 Survival estimates

Survival Criteria	Arm/Cohort	Patients, No.	Median Survival, Mo	Range,* Mo	*P* Value
Survival from date of progression after vaccination	A	18	7.40	3.37-16.70	.065
	B	20	2.73	2.03-7.20	
	C	31	3.23	1.97-7.53	
Survival from date of study enrollment	A	18	12.18	9.27-18.33	.313
	B	20	6.32	5.13-13.93	
	C	31	6.20	4.17-11.57	
Survival based on immune response	Negative	30	9.28	6.20-15.00	.250
	Positive	13	9.23	4.73-NA	

*95% confidence interval
